# An Inquiry into the Efficacy of Digitalis in the Treatment of Idiopathic Epilepsy

**Published:** 1841-04-01

**Authors:** 


An Inquiry into the Efficacy of Digitalis in the Treatment of Idiopathic Epilepsy. By *Edmond Sharkey,* A.B. M.D. one of the Lecturers on Midwifery in the Hunterian School of Medicine, Charlotte Street. 8vo. pp. 80. Highley, London, January, 1841.

Most practitioners are aware of the obstinate nature and the deplorable consequences of epilepsy. Yet, every now and then we find it disappear, with or without remedies. Where there is no actual deformity of the skull, or imbecility of the mind, we ought to give a fair trial to medicine and diet. Whatever specific we may employ, a regulation of all the great functions is the primary and the principal object. This procedure alone, will sometimes arrest the disease or greatly prolong the intervals between the attacks. The next prominent indication is a drain, by means of a seton, from the neighbourhood of the head. These failing, recourse must be had to nostrums or specifics, as the nitrate of silver, the oxide of zinc—or, lastly, digitalis, the subject of the treatise.

We are informed by Dr. Sharkey, that about nine years ago, his father published a paper in the Lancet on the Use of Digitalis in Epilepsy—but no notice has since been taken of the remedy by any authors. Some of these cases are re-stated by Dr. Sharkey, and others are added. We shall condense some of the more interesting of these.

*Case* 1.—Miss Fowkes, aged 17 years, had been epileptic for twelve months, and commenced the present plan on the 4th Sept. 1817.

℞. Fol. digital. purp. recent. ℥iiss. contunde in mortario in pulpam; deinde adde cerevisiæ fortioris Oj. infunde per horas septem, deinde cola exprimendo. Capt. liquoris colati ℥iv. cum pulv. fol. Polypodii quercus siccatorum, aut radicis siccatæ gr. x.

5th Sept.—In ten minutes after taking the draught yesterday, she had a fit, but shorter and less severe than usual. She vomited frequently and violently till twelve o'clock to-day—pulse sunk from 120 to 54, intermitting and irregular—great headache and pain in the epigastrium—cramps in the legs—extremities cold. 6th. Had no fit—vomiting continues till ten A.M. to-day—pulse 40, irregular—great prostration of strength—pupils dilated from the beginning. 7th. Continues to vomit bilious matters—pulse 40—fæces bilious. 8th. Continues free from fits—vomits much—pulse 60, irregular—great debility—pupils dilated. 16th. Continues free from fits—strength restored—pupils contract—appetite good. Our author watched this case for two years, and there was no return of the fits.

There is no description of this young lady's fits, but from some expressions, they appeared to have been almost daily, before the Herculean remedy was applied. This is not the character of epilepsy. We have little doubt that the case was one of those anomalous hysterical convulsions to which many females are subject. But whatever was the nature of the malady, we think the patient was as nearly destroyed by the remedy as any could possibly be. We should be extremely sorry to exhibit such a dose to a young female, or, indeed, to any one.

*Case* 2.—A gentleman had been afflicted with epilepsy for twenty years. He was of robust form, sanguine temperament, great abdominal congestion, but general good health.

"He was treated by several physicians of eminence, and pronounced incurable. Amongst other remedies, he took nitrate of silver, to a large amount, which discoloured him horridly, without advantage. Suffice it to say, for the sake of brevity, that I had him under preparatory * treatment for two or three months without any abatement of the fits. I administered the digitalis and polypody (the leaves) to him as above, with similar effects. The prostration of strength and diminution of pulse in this case was absolutely frightful. He has had no return of fits for ten years.†

I also cured his brother six years ago, who likewise has been since exempt." 22.

*Case* 3.—This patient was insane as well as epileptic. When the dose was given, the pulse was 140. He refused to drink gruel after the dose, (which, we observe, was generally given,) and did not vomit till the next day, and that by the assistance of ipecacuan. His pulse had fallen to 56. He was removed to a lunatic asylum, where, it is said, he recovered his reason, and remained free from fits.

In two or three other cases of epilepsy complicated with uterine derangement, the treatment failed in the hands of Dr. Percival.

In 1827, Mr. Scott published a case of hydrocephalus in a boy, nine years of age, accompanied by epileptic fits. The hydrocephalus was removed, but the fits continued to recur daily, and usually six or seven times in the twenty-four hours.

"They were generally preceded by twitchings of the facial muscles, but sometimes gave no indication of their approach. Leeches and blisters to the head were first tried, but with the effect of rendering the fits more frequent and severe. He then was put upon the use of Tinct. Digital. which was gradually increased so far as gtt. xii. three times daily. Under this treatment a cessation of the fits for four months was obtained, but the twitches continued. The digitalis was now stopped, and the fits returned; but were milder, and recurred once in ten days. The mother of the boy having observed that the twitches were more violent at the approach of the fit, adopted the plan of giving the Tinct. Digital. freely when these were observed; thus the twitchings were diminished, and the fits apparently prevented. She sometimes gave as much as a teaspoonful at once. On one occasion the boy got at the bottle, and drank the entire contents, (from two to three drachms,) producing the usual symptoms of an over-dose, which were removed by the administration of brandy and opium. He had two severe fits at the time. The mother's account was, that an amendment commenced after this." 26.

The final issue of this case is not stated; but it is perfectly clear that these fits were convulsive rather than epileptic.

A case is related in our Edinburgh contemporary, in which "fits of im-

---

* "Regulation of secretions, &c."

† "In reply to inquiries made of this gentleman, through my friend Dr. Wood, of Cork, I yesterday was informed 'that he never had them after; and that he also had an acquaintance who was subject to them, who was treated by my father with digitalis, and recovered."

perfect epilepsy co-existed with diseased heart, for a period of three years and a half." Digitalis, in doses of a quarter of a grain, was given, and when two grains were taken the fits disappeared. The case, however, is not very clearly stated.

*Case* 4.—There is a case published in the Lancet, October 8th, 1831, by Mr. A. Courtney, where a boy was seized with epilepsy at the age of 16 years, without assignable cause. He continued subject to fits for nine years, and, in the year 1802, took digitalis and polypody, in the following manner.

"'Recent leaves of digitalis four ounces, infused for twenty-four hours in a pint of boiling water. When strained, this was divided into three doses, one of which was ordered to be taken every third day with fifteen grains of the dried leaves of polypody in powder. But such was the effect of the first dose, that his relations would not permit him to take a second; for, a few minutes after he had taken it, a vomiting commenced, which, in spite of every thing that could be done to allay it, continued almost incessantly for five days, accompanied with such prostration of strength, that it was thought at times doubtful whether he was dead or alive.' He never had a return of the fit up to the date of the communication." 28.

It is needless to advert to any more of these cases, because they appear to us very unsatisfactory, and as almost all of them have been already published. The author institutes a comparison between the success of digitalis, and that of some other heroic remedies, especially the oil of turpentine and the nitrate of silver. It need hardly be stated that the comparison is in favour of the remedy proposed by himself. But it requires not the gift of prophecy to foretel that this remedy will never come into general use. Digitalis, in the moderate doses of common practice, can be of no service in epilepsy—and in the doses recommended in this work, it would be dangerous, especially as it is a very uncertain medicine—some constitutions bearing it well, while to others it proves a poison. This uncertainty is far greater than with any other medicine in the Pharmacopœia. It is a very cumulative drug, sometimes appearing to lie dormant for a time in the system, till an explosion of alarming symptoms takes place, which, if not dangerous, are extremely distressing. The evidence in favour of digitalis does not appear to us so great as it does to Dr. Sharkey. We think the balance is far on the side of the nitrate of silver. The great objection to the latter—the discolouration of the skin, is now pretty well removed by the fact, nearly, if not completely, ascertained, that the oxide of silver does not produce the same phenomenon. The nitrate or oxide of silver never injures the stomach, but, on the contrary, proves one of the most powerful antidyspeptic remedies we possess. We have never met with a single instance of discolouration, where the exhibition of the nitrate was limited to three months. Dr. Sharkey never alludes to this immunity, though promulgated by the late Dr. Baillie, and others, long ago. Dr. S. seems to hint, at page 48, that the discolouration is "*an injury extending to their offspring*." If Dr. S. can adduce an instance of this transmission of blue skin to the progeny, we shall be exceedingly surprized. We should just as soon expect to see the children of Greenwich and Chelsea pensioners born with wooden legs.

The greater part of Dr. S.'s work is occupied with physiological discussions respecting the operation of medicines, and especially of digitalis, which are really very ingenious, though not always conclusive, and indicate a talented and well-stored mind. We have only room for one extract from this part of the work.

"*Precautions.*—As fatal results have occurred in persons who were for the cure of other diseases put under the full influence of digitalis, from want of proper caution, it seems necessary to state here that a patient, under these circumstances, should on no account be allowed to assume the erect posture, for, in the instances alluded to, (which however occurred in persons labouring under debilitating diseases,) the first effect of such effort was to increase the rapidity and diminish the power of the circulation to such an extent as to produce, first, fainting, and subsequently death. And although I never saw or heard of any such accident in an epileptic so treated, and although, from its being a disease of unnatural excitability, I should say à priori that it was not likely to take place, yet the precaution is a proper one, and should in no case be omitted; more especially when we remember that in general the recumbent posture is less favourable to the epileptic invasion than the erect.* It is also better not to interfere with the course of its operation by the use of any stimulants, unless they are imperatively called for; should the necessity, however, arise, the means recommended in such cases should be put in practice. If, while the alarming train of symptoms mentioned as the effects of poisonous doses, (cold sweats, delirium, repeated faintings, convulsions, local and general,) have set in without the previous occurrence of vomiting; and if we fail in our endeavours to excite this by emetics, titillation of the fauces, &c. the stomach-pump should be used, ammonia and brandy administered, and in such cases, above all others, the horizontal posture strictly enforced. But I must again repeat, that the extreme case now supposed, is drawn purely from imagination, so far as my own experience of large doses of digitalis in this disease is concerned." 73.

Notwithstanding our dissent from the author on some practical, and even doctrinal points, we recommend his work to our readers as well worthy of perusal.

* This is surely an oversight of Dr. Sharkey's. Ten fits of epilepsy take place in the recumbent, for one in the perpendicular posture.—(Ed.)

